# Capacity of Public Health Surveillance to Comply with Revised International Health Regulations, USA

**DOI:** 10.3201/eid1605.091127

**Published:** 2010-05

**Authors:** Kia E. Armstrong, Scott J. N. McNabb, Lisa D. Ferland, Tim Stephens, Anna Muldoon, Jose A. Fernandez, Stephen Ostroff

**Affiliations:** Centers for Disease Control and Prevention; Atlanta, Georgia, USA (K.E. Armstrong, S.J.N. McNabb); Council of State and Territorial Epidemiologists, Atlanta (L.D. Ferland); US Department of Health and Human Services, Washington, DC, USA (T. Stephens, A. Muldoon, J.A. Fernandez); Pennsylvania Department of Health, Harrisburg, Pennsylvania, USA (S. Ostroff)

**Keywords:** World Health Organization, population surveillance, reporting capacity, International Health Regulations 2005, United States, research

## Abstract

Public health surveillance is essential for detecting and responding to infectious diseases and necessary for compliance with the revised International Health Regulations (IHR) 2005. To assess reporting capacities and compliance with IHR of all 50 states and Washington, DC, we sent a questionnaire to respective epidemiologists; 47 of 51 responded. Overall reporting capacity was high. Eighty-one percent of respondents reported being able to transmit notifications about unknown or unexpected events to the Centers for Disease Control and Prevention (CDC) daily. Additionally, 80% of respondents reported use of a risk assessment tool to determine whether CDC should be notified of possible public health emergencies. These findings suggest that most states have systems in place to ensure compliance with IHR. However, full state-level compliance will require additional efforts.

## Abstract 2

Most states have the reporting capacity to comply.

The 2005 revisions to the International Health Regulations (IHR 2005) were a major global policy achievement to ensure international recognition and notification of unusual public health events. These regulations are an international legal instrument that binds 194 countries (World Health Organization [WHO] member states). The goal of IHR 2005 is to help the international community prevent or respond to acute public health risks that have the potential to cross borders and threaten the global population. As seen with the emergence of pandemic (H1N1) 2009, diseases have the potential to spread quickly around the globe through international travel and trade (*1*). Member states are required to report certain diseases and public health events to WHO. Furthermore, the rights and obligations of member states are defined to establish procedures that WHO must follow to uphold global public health security (*2*).

The 2005 revision was the first major update to the IHR since 1969 and was designed to reflect trends in disease emergence and spread over the past several decades. The 2005 revision also was meant to unify the considerable changes in communication capacity, disease surveillance, and investigation infrastructure. Member states must report potential public health emergencies of international concern (PHEIC), including those of biologic, chemical, radionuclear, or unknown origin, to WHO. A common decision matrix that focuses national reporting around a risk assessment process is used rather than sole reliance on reporting of specific diseases or incidents (*3*). This method of reporting requires all member states to develop, strengthen, and maintain a core set of public health surveillance and response capacities at the local, intermediate, and national levels (*4*).

After IHR revision in 2005, member states were provided a 2-year window in which to assess their surveillance and response capacities, focusing on 4 necessary characteristics of surveillance systems: timeliness, sensitivity, stability, and usefulness (*3*). Each of the 194 member states also was required to designate a National Focal Point that would assess any event within 48 hours. After the assessment specified in Appendix 2 of IHR 2005, each member state must notify WHO of any potential PHEIC. Therefore, core public health surveillance systems at local and national levels must be capable of ensuring national awareness of incidents in a timely manner.

Surveillance systems exist at many levels: clinics; hospitals; and local, state, national, regional, and global levels. To be effective, these different levels must be well integrated. Literature on surveillance systems often examines each level separately or, at most, the links between 2 adjacent levels, describing the importance of integrating each system so that communication between levels is more effective (*3*,*5*–*7*). State-to-national notification is a key aspect of federalist systems and has been viewed as a key challenge for countries with this type of government structure (*3*); several authors have noted the political and practical difficulties these surveillance systems may face and the various ways national disease surveillance can be facilitated (*8*).

In the United States, reporting of nationally notifiable diseases to the Centers for Disease Control and Prevention (CDC) by states is voluntary. Public health surveillance takes place within a state on the basis of reports received from a variety of sources, typically local. Reporting is mandated by state legislation or regulation. States then determine whether CDC should be notified. Notifications from states, territories, and the District of Columbia are collected and analyzed by the National Notifiable Diseases Surveillance System. A 2004 review of this system showed that for meningococcal disease laboratory results, local entities reported to their states and then states notified NNDSS within 2–117 days (*5*). More recently, 60% of meningococcal diagnosis reports were received by states within 1 day after diagnosis (*9*).

The literature identifies essential elements that surveillance systems need to meet IHR 2005 criteria, including electronic information systems and supportive infrastructure, to ensure timely reporting to the National Focal Point (*6*,*7*). In addition, intergovernment cooperation with both formal and informal communication, from the local to the international level, are essential aspects of successfully functioning public health surveillance systems (*3*,*6*,*8*) and can ensure rapid reporting of incidents before laboratory confirmation is received (*3*,*6*). Overall, key aspects of successful surveillance systems identified in the literature align closely with the requirements of IHR 2005. This alignment suggests that systems built in accordance with the agreement will provide successful global coverage.

Since the new regulations took effect, no studies have been done to determine the timeliness of reporting conditions specified in IHR 2005. In addition, no reports exist that discuss timeliness of notification to CDC about unusual cases or outbreaks of unknown cause. To address these gaps and to determine the ability of states to comply with IHR 2005, we assessed state surveillance capacities through surveys completed by the Council of State and Territorial Epidemiologists (CSTE). This assessment examined several key requirements that are necessary to effectively meet national responsibilities and ensure compliance with IHR 2005.

## Methods

In February 2009, CSTE electronically distributed a structured, self-administered questionnaire to state epidemiologists in all 50 states and Washington, DC. Responses were made anonymous at the time of data analysis. The questionnaire was designed to address the following questions: 1) How are states able to determine the status of potential public health emergencies? 2) Are local health departments able to report in a timely manner to the correct point-of-contact (POC) in their state? 3) Are states able to notify CDC of public health emergencies in a timely manner (i.e., within 24 hours)? 4) Do states support or implement other control measures (i.e., collaboration with other departments or cross-jurisdiction)? Frequencies and percentages were used to describe the results.

The CSTE State Reportable Conditions Assessment, completed by state epidemiologists, also was used to assess state reporting practices. Information was obtained from the 2007 Assessment, which is available for review by all states and territories (*10*).

## Results

**Figure 1:**
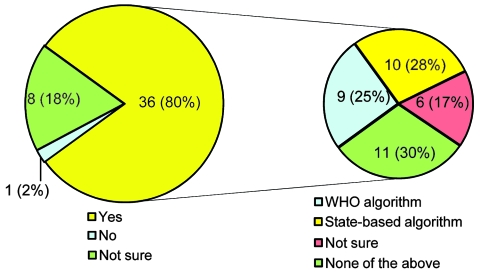
Proportion of state epidemiologists who use risk assessments to determine whether notification to the Centers for Disease Control and Prevention is necessary, showing types of algorithms used, United States, 2009. WHO, World Health Organization.

A total of 47 (92%) of the 51 eligible jurisdictions responded to the questionnaire. Eighty percent of respondents reported the use of risk assessments to determine the necessity of notifying CDC about unusual or unexpected events (Figure 1). Of those who used risk assessments, ≈50% used them to initiate formal investigations. About 50% of respondents reported the use of risk assessments to evaluate whether notification to the state health officer (51%), chief emergency response/management office (47%), and CDC or other federal entities (51%), respectively, was necessary. Twenty-eight percent reported use of a state-based algorithm; 25% reported use of the WHO algorithm for risk assessments. The remainder of respondents used another algorithm (excluding a state-based algorithm or the WHO algorithm) or were not sure of the algorithm used in their state.

More than 90% of jurisdictions required reports of suspected and probable cases of the 4 immediately notifiable IHR 2005 conditions (i.e., smallpox, poliomyelitis caused by wild-type poliovirus, human influenza caused by a new subtype, and severe acute respiratory syndrome [SARS]) within 24 hours after diagnosis (Table 1). In addition, 96% of states reported that they would notify CDC of suspected and probable cases of IHR 2005 conditions within 24 hours (Table 1). Eighty-one percent of respondents reported having the capacity to transmit daily notifications to CDC. Of those unable to transmit daily notifications, 5 indicated that they would be able to provide daily electronic data to CDC within <1 year

**Table 1:** Reported circumstances and time frames for reporting and notification of International Health Regulations conditions by state epidemiologists, USA, 2009*

Circumstance	Immediately, no. (%)	Within 4 h, no. (%)	Same business day, no. (%)	Within 24 h, no. (%)	Total no.
Novel influenza virus					
Time frame for reporting					
Suspected	23 (52)	2 (5)	1 (2)	5 (11)	37
Probable	1 (2)	0	0	1 (2)	4
Total†	25	2	1	6	44
Time frame for notification					
Suspected	16 (36)	9 (20)	3 (7)	1 (2)	29
Probable	9 (20)	0	3 (7)	1 (2)	14
Confirmed	0	0	1 (2)	0	1
Total†	26	9	7	2	45
Severe acute respiratory syndrome					
Time frame for reporting					
Suspected	25 (56)	1 (2)	4 (9)	8 (18)	41
Probable	1 (2)	1 (2)	0	0	2
Confirmed	0	0	0	1 (2)	1
Total†	27	2	4	9	45
Time frame for notification					
Suspected	16 (36)	9 (20)	4 (9)	2 (4)	32
Probable	8 (18)	1 (2)	3 (7)	0	12
Confirmed	1 (2)	0	0	0	1
Total†	25	10	7	2	45
Smallpox					
Time frame for reporting					
Suspected	30 (67)	2 (4)	1 (2)	6 (13)	41
Probable	0	1 (2)	0	0	2
Confirmed	0	0	0	1 (2)	1
Total†	31	3	1	7	45
Time frame for notification					
Suspected	23 (51)	5 (11)	4 (9)	1 (2)	33
Probable	8 (18)	2 (4)	0	0	11
Total†	32	7	4	1	45
Poliomyelitis, wild type					
Time frame for reporting					
Suspected	22 (49)	1 (2)	1 (2)	9 (20)	37
Probable	2 (4)	2 (4)	0	0	4
Confirmed	0	0	0	1 (2)	3
Total†	25	3	1	10	45
Time frame for notification					
Suspected	11 (24)	9 (20)	5 (11)	1 (2)	27
Probable	8 (18)	1 (2)	3 (7)	2 (4)	14
Confirmed	1 (2)	0	0	1 (2)	3
Total†	21	10	8	4	45

*n = 45 for all percentages except time frame for reporting of novel influenza virus (n = 44). †Includes states that did not have a specified time for reporting and/or notification or they were not sure of the time frame for reporting and/or notification.

**Figure 2:**
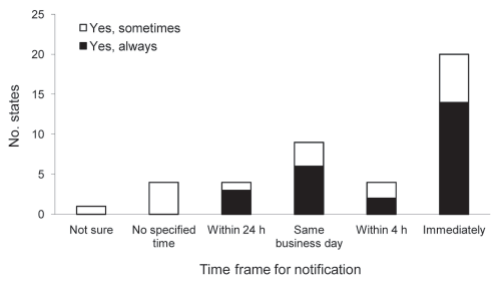
Number of states that notify the Centers for Disease Control and Prevention of an unusual or unexpected case or outbreak of disease, by time frame, United States, 2009.

All respondents reported they would either always or sometimes notify CDC of an unusual or unexpected case or outbreak; 60% reported they would always notify CDC within 24 hours (Figure 2). Among the respondents, 30% would sometimes notify CDC within 24 hours. According to the 2007 State Reportable Conditions Assessment, 48 of the jurisdictions included unusual or unexpected events on their reportable conditions lists.

**Figure 3:**
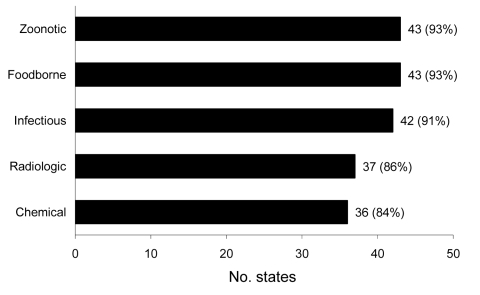
Number of state epidemiologists who have points-of-contact within the state for reporting different types of potential public health emergencies of international concern, United States, 2009.

Furthermore, most states could identify a specific POC in their state for reporting various public health events and emergencies (Figure 3). Ninety-one percent of states reported having a designated POC for zoonotic, foodborne, and infectious events; for chemical and radiologic events, 84% and 86% of states, respectively, reported having a designated POC.

More than 50% of respondents reported having formal information-sharing systems or mechanisms pertaining to emergencies or outbreaks with state law enforcement, emergency management and homeland security, agriculture, environmental protection, and fish and wildlife agencies, excluding the state departments of transportation (37%) (Table 2). Fifty-one percent reported participating in cross-jurisdiction electronic surveillance and having reporting systems for foodborne and infectious diseases with neighboring states.

**Table 2:** Proportion of state agencies that have formal information-sharing systems or mechanism for emergencies or outbreaks within state government, USA, 2009

Government agency	No./total reporting (%)
State law enforcement	33/44 (75)
Emergency management and/or homeland security	37/44 (84)
Agriculture	35/45 (78)
Transportation	17/44 (37)
Environmental protection	30/44 (68)
Fish and wildlife	28/45 (62)

## Discussion

Most states can successfully conduct public health surveillance in compliance with IHR 2005. However, full state-level capacity for compliance was not found on any assessment response. Additional efforts are needed to ensure the ability of the United States to meet its IHR 2005 obligations.

Most states reported use of risk assessments to determine the need to notify CDC about unusual or unexpected events. In addition, ≈50% of states reported the use of risk assessment when initiating a formal investigation. IHR 2005 emphasizes the use of risk assessments to notify WHO about public health emergencies, rather than about specific events. The use of risk assessments in notifying CDC can help determine whether notification is necessary and ensure timely notification without waiting for laboratory confirmation.

Most (>80%) states reported having 1 POC for reporting chemical, radiologic, foodborne, infectious, and zoonotic events. All states should have a POC to facilitate prompt assessment and appropriate reporting. Such contacts also can assist in assessing events outside their areas of expertise or with unknown cause.

Reports of capacity to transmit daily notifications to CDC suggest that data collection and transmission capacity has improved substantially in recent years. The ability to transmit reports to CDC is a critical function, which allows for national situational awareness in high-profile events and public health emergencies.

Not all respondents included unusual or unexpected events on their state’s reportable conditions lists. Adding this criterion would help ensure that conditions having the potential to become public health emergencies can be recognized and reported in a timely manner.

Internal and external relationships of each state can play a role in reporting. Most states have formal information-sharing systems with other agencies within their state. Such dissemination of information within a state increases the likelihood that IHR reportable events are appropriately evaluated and reported. Other agencies may have knowledge that could be incorporated into a risk assessment.

About 50% of respondents reported participation in cross-jurisdictional electronic surveillance and having reporting systems for foodborne and infectious diseases. Public health surveillance across state jurisdictions is as important as the sharing of information within a state. Neighboring states can be alerted to public health emergencies that have the potential to become widespread or even global.

Our assessment has several limitations. First, only states were assessed; neither territories nor local health departments were included. Circumstances are sufficiently different in the territories; no conclusions about their capacity should be drawn because they are likely to have different reporting practices and capacities. Future assessments should focus on identifying and documenting capacities for IHR 2005 compliance in at least a sample of jurisdictions at the local or county level. Second, our assessment relied on self-reporting, which in some instances may have led to inaccuracies or bias. Data were made anonymous before analysis so that states would not be singled out as having suboptimal reporting practices or capacities. Therefore, we could not inquire about potential discrepancies.

To enable the United States to fully meet its IHR 2005 obligations, all states should include unusual or unexpected events or outbreaks on their state lists of reportable conditions. In addition, states and CDC should work toward further development of the nationally notifiable conditions list and the timeframes for reporting. The capacity to transmit records to CDC on a daily basis is key to full compliance with IHR 2005. Risk assessments of unusual or unexpected events should be performed to determine whether they meet requirements for notification to CDC as a potential PHEIC. Performing such risk assessments will enable timely notification to CDC, even before laboratory confirmation. Furthermore, state POCs are likely to facilitate recognition and reporting of potential public health emergencies within their respective states.

State health departments should work to ensure that their counterparts in state government and in local health departments understand the requirements of IHR 2005; reporting exercises may help accomplish this goal. All health officials, particularly those at the state level, should have a basic understanding of these international regulations, especially the reporting and notification timeframes and practices. Reporting exercises would give state and local health officials the ability to assess potential public health emergencies in a practice environment and allow a broader perspective of when notification is necessary.

Expansion of cross-jurisdictional surveillance and reporting systems also would benefit national recognition and investigation of public health emergencies, especially for foodborne illness and infectious diseases. Such systems are not explicitly required by IHR 2005 but would assist states in the assessment and timely reporting of public health emergencies, both of which are necessary for compliance. National, state, and local government agencies should assist states in implementing these practices and developing appropriate infrastructures.
